# Differences in the flavonoid composition of the leaves, fruits, and branches of mulberry are distinguished based on a plant metabolomics approach

**DOI:** 10.1515/biol-2022-0886

**Published:** 2024-06-27

**Authors:** Yewei Zhong, Fenglian Tong, Junlin Yan, Huiwen Tan, Adalaiti Abudurexiti, Rui Zhang, Yi Lei, Delong Li, Xiaoli Ma

**Affiliations:** College of Pharmacy, Xinjiang Medical University, Urumqi 830011, China

**Keywords:** mulberry, flavonoid metabolites, UPLC–ESI-MS/MS

## Abstract

Mulberry is a common crop rich in flavonoids, and its leaves (ML), fruits (M), and branches (Ramulus Mori, RM) have medicinal value. In the present study, a total of 118 flavonoid metabolites (47 flavone, 23 flavonol, 16 flavonoid, 8 anthocyanins, 8 isoflavone, 14 flavanone, and 2 proanthocyanidins) and 12 polyphenols were identified by ultra-performance liquid chromatography–electrospray ionization-tandem mass spectrometry. The most abundant in ML were 8-*C*-hexosyl-hesperetin *O*-hexoside and astragalin, the most abundant in M were 8-*C*-hexosyl-hesperetin *O*-hexoside and naringenin, and the most abundant in RM were cyanidin 3-*O*-galactoside and gallocatechin–gallocatechin. The total flavonoid compositions of ML and RM were essentially the same, but the contents of flavonoid metabolite in more than half of them were higher than those in M. Compared with ML, the contents of flavone and flavonoid in RM and M were generally down-regulated. Each tissue part had a unique flavonoid, which could be used as a marker to distinguish different tissue parts. In this study, the differences between flavonoid metabolite among RM, ML, and M were studied, which provided a theoretical basis for making full use of mulberry resources.

## 1 Introduction

Mulberry (*Morus alba* L.) was first recorded in the traditional Chinese Medical Book *Shennong’s Herbal Classic of Materia Medica*, and its leaves (ML), fruits (M), and branches Ramulus Mori (RM) can be used as medicine. Mulberry leaves are often used in the treatment of cardiovascular and cerebrovascular diseases or alopecia [1]. Mulberry is a kind of fruit in daily life. It is often used to protect the kidney and liver and reduce sugar and fat [2] and is called “Folk Holy Fruit.” Mulberry branches are often used in the treatment of arthritis and rheumatism [3]. Flavonoids constitute a major group of important secondary plant metabolites. The group includes representatives of anthocyanins, flavonoids, and chalcone. They have been reported to have a variety of biological functions in the plants themselves and have shown impressive antioxidant, anti-inflammatory, hypoglycemic, lipid-lowering, and antihypertensive effects in the human organism [4]. Flavonoids play an important role in the bioactive components of mulberry, and different parts of mulberry have different medicinal characteristics, and the differences in their flavonoid metabolites may have an important influence on this [5]. Therefore, exploring the differences of flavonoid metabolites in different parts of mulberry and revealing the possible basis of medicinal components will be an important revelation for the study of the medicinal development of mulberry.

Mulberry-specific compound 1-deoxynojirimycin has a significant hypoglycemic effect, the group’s previous study found that 1-deoxynojirimycin is the highest content in leaves, followed by the second in fruits, and the lowest in stems, which provides some reference significance for the application of mulberry research. However, there is no study on the difference in its flavonoid components, to further explore the basis of the substance active components of mulberry this study will use the metabolomics approach to fill this gap. Metabolomics of mulberry has been widely studied and reported, Jiang et al. analyzed the changes in the content of different sugar and acid components of mulberry fruits from the green stage of fruit expansion to the red and then to the ripening stage based on targeted metabolomics by LC-MS [6]. Yang et al. investigated the effect of frost on the different metabolites of two types of mulberry leaves (*Morus nigra* L. and *Morus alba* L.) [7]. In this study, metabolomics will be utilized to explore the differences in the bioactivity of flavonoids in different parts of mulberry, which will provide valuable insights into the uses of their different parts. Ultra performance liquid chromatography–electrospray ionization-tandem mass spectrometry (UPLC–ESI-MS/MS) combined with cluster analysis, principal component analysis (PCA), and orthogonal partial least squares-discrimination analysis (OPLS-DA) were used to analyze the difference of flavonoids metabolites in ML, RM, and M.

## 2 Materials and methods

### 2.1 Plant materials

The three medicinal parts of mulberry were harvested from Hotan, Xinjiang, in July 2020 and identified as mulberry leaves, mulberry, and mulberry branches by Professor Haiyan Xu, College of Traditional Chinese Medicine (TCM), Xinjiang Medical University; the method of identification is based on the original identification of TCM [8]. All parts were shade-dried, air-dried, crushed through a 100-mesh sieve, and stored in the College of Pharmacy of Xinjiang Medical University (humidity at 65–70% and temperature at 4℃). Nine samples were selected in this metabolic group study and divided into three groups: stem, leaf, and fruit. Each group had three biological replicates (Table 1).

**Table 1:** Sample grouping

Tissue position	Processing description	Sample name	Group
Mulberry leaves	Drying and pulverizing	ML1	ML
Mulberry leaves	Drying and pulverizing	ML2	ML
Mulberry leaves	Drying and pulverizing	ML3	ML
Fruit of mulberry	Drying and pulverizing	M1	M
Fruit of mulberry	Drying and pulverizing	M2	M
Fruit of mulberry	Drying and pulverizing	M3	M
Mulberry branch	Drying and pulverizing	Ramulus_Mori_1	RM
Mulberry branch	Drying and pulverizing	Ramulus_Mori_2	RM
Mulberry branch	Drying and pulverizing	Ramulus_Mori_3	RM

### 2.2 Standard products and reagents

The standard products used in the self-built library are as follows: Yunnan Xili Biotechnology Co., Ltd (http://www.biobiopha.com/) and Sigma Aldrich (Shanghai) Trading Co., Ltd (Sigma-Aldrich); methanol, acetonitrile, and ethanol (Merck, Germany) are chromatographic grade. The standard product was dissolved with dimethyl sulfoxide (Merck, Germany) or methanol as solvent and stored at −20℃. Before mass spectrometry, 70% methanol was diluted to different gradient concentrations.

### 2.3 Sample preparation and extraction

The sample was freeze-dried in a vacuum; ground (30 Hz, 1.5 min) to powder by grinding instrument (MM 400, Retsch); weighed 100 mg powder; and dissolved in 1.0 mL extraction solution (70% methanol–water solution); the dissolved samples were refrigerated overnight at 4℃, during which they were vortexed three times to improve the extraction rate; after centrifugation (rotating speed: 10,000 × *g*, 10 min), absorb the supernatant and use microporous filter membrane (0.22 μm) The samples were filtered and stored in the injection bottle for UPLC–MS/MS analysis.

### 2.4 UPLC conditions

Chromatographic column: Waters ACQUITY UPLC HSS T3 C18 (1.8 μm, 2.1 mm × 100 mm); column temperature: 40℃; mobile phase: ultra-pure water (0.04% acetic acid) as phase A and acetonitrile (0.04% acetic acid) as phase B; gradient elution procedure: 0 min, 5% B; 0–11.0 min, 5–95% B; 11.0–12.0 min, 95% B; 12.0–12.1 min, 95–5% B, 12.1–15.0 min, 5% B; flow rate 0.4 mL/min; the injection volume was 2 μl; internal standard: l-2-chlorophenylalanine (China, J&K Scientific).

### 2.5 Mass spectrum condition

Positive and negative ion ionization modes were used for mass spectrometry detection, and the scanning range was *m/z* 100–2,000. The specific parameters are shown in Table 2.

**Table 2:** Mass spectrometry

Mass spectrometry conditions	Parameter
Mass spectrometry voltage	5,500 V
Collision-activated dissociation	High
Collision energy	Specific optimization
Electrospray ionization temperature	500℃
Curtain gas	25 psi
Declustering potential	Specific optimization

### 2.6 Qualitative and quantitative analyses of metabolites

Based on the self-built database metware database (China Wuhan Jiewei Biotechnology Co., Ltd.) [9] and the public database of metabolite information, the first and second data of mass spectrometry were qualitatively analyzed. HMDB is used for structure analysis (http://www.hmdb.ca/) and METLIN (http://metlin.scripps.edu/index.php). Metabolite quantification was performed by multiple reaction monitoring (MRM) mode analysis of triple quadrupole mass spectrometry. In MRM mode, the quadrupole first selects the precursor ion (parent ion) of the target material and eliminates the corresponding ions of other molecular weight materials to preliminarily eliminate interference; the precursor ion is induced to ionize by the collision chamber and breaks to form a lot of fragment ions, and then the fragment ions are filtered by the triple quadrupole to select a required characteristic fragment ion and eliminate the interference of non-target ions, making the quantification more accurate and repeatable. After obtaining the mass spectrum analysis data of metabolites from different samples, the peak areas of all mass spectrum peaks were integrated, and the mass spectrum peaks of the same metabolite in different samples were integrated and corrected.

### 2.7 Statistical analysis

Each group had three biological replicates. Hierarchical cluster analysis, PCA, and OPLS-DA were performed by R 3.2.5 software. Adobe Photoshop CC 2019 was used for image beautification, and the Kyoto Encyclopedia of Genes and Genome (KEGG) (http://www.genome.ad.jp/kegg/) database was used for path enrichment analysis.

## 3 Result

### 3.1 Sample quality control (QC) analysis

**Figure 1:**
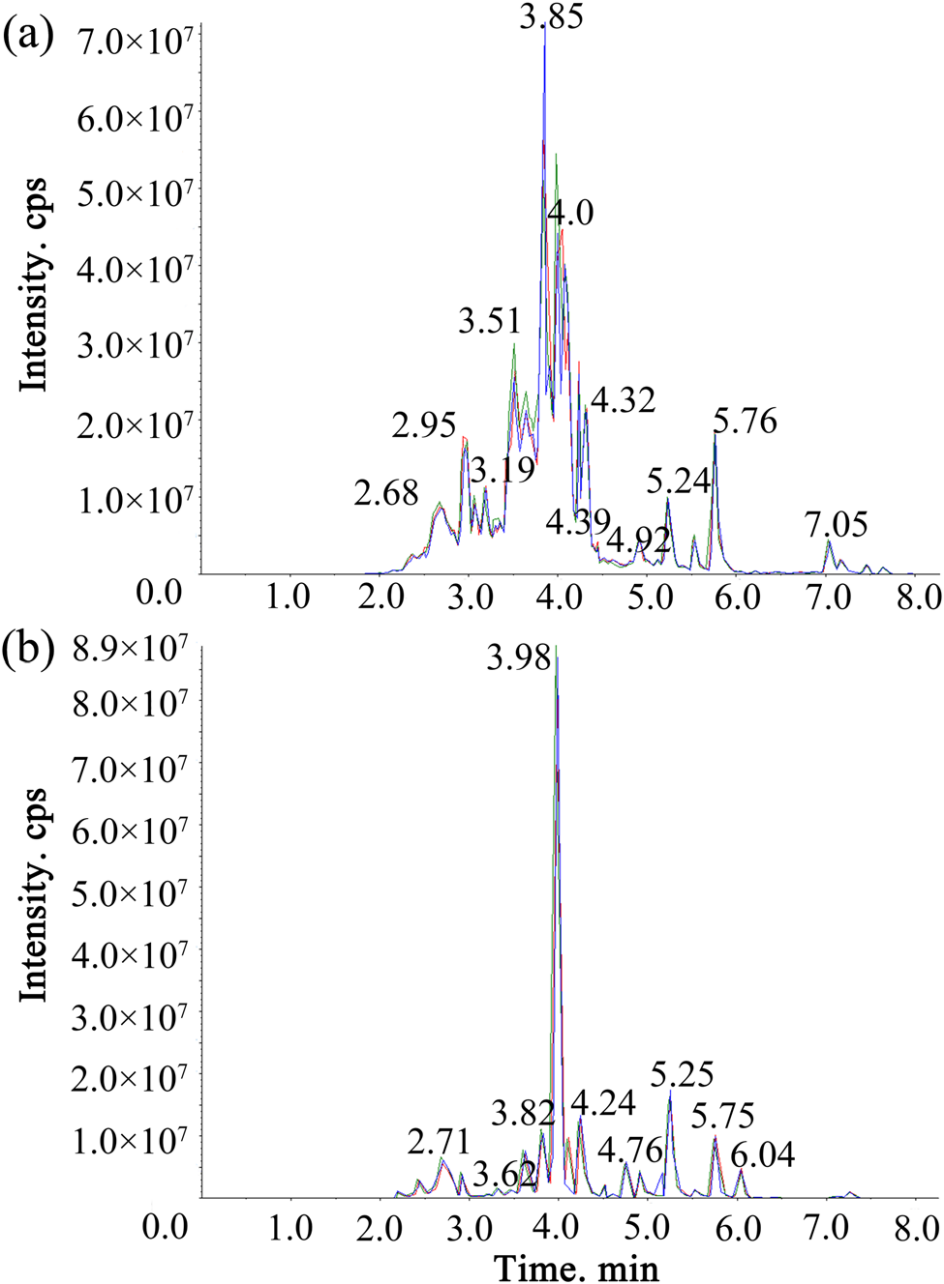
The stacking diagram of TIC maps from QC samples mass spectrometry. (a) TIC of positive ion MRM. (b) TIC of negative ion MRM.

The high stability of the instrument provides an important guarantee for the repeatability and reliability of the data. The three groups of sample extracts were mixed to prepare QC samples, and the reproducibility of samples under the same treatment method was analyzed. The reproducibility of metabolite extraction and detection can be judged by overlapping analysis of total ion current diagrams of different QC samples identified by mass spectrometry. The overlay of the total ions current (TIC) diagram is shown in Figure 1. The results showed that the total ion current curve of metabolite detection had a high degree of overlap, indicating that the signal stability of the same sample was good at different detection times.

### 3.2 Metabolic profiling

**Figure 2:**
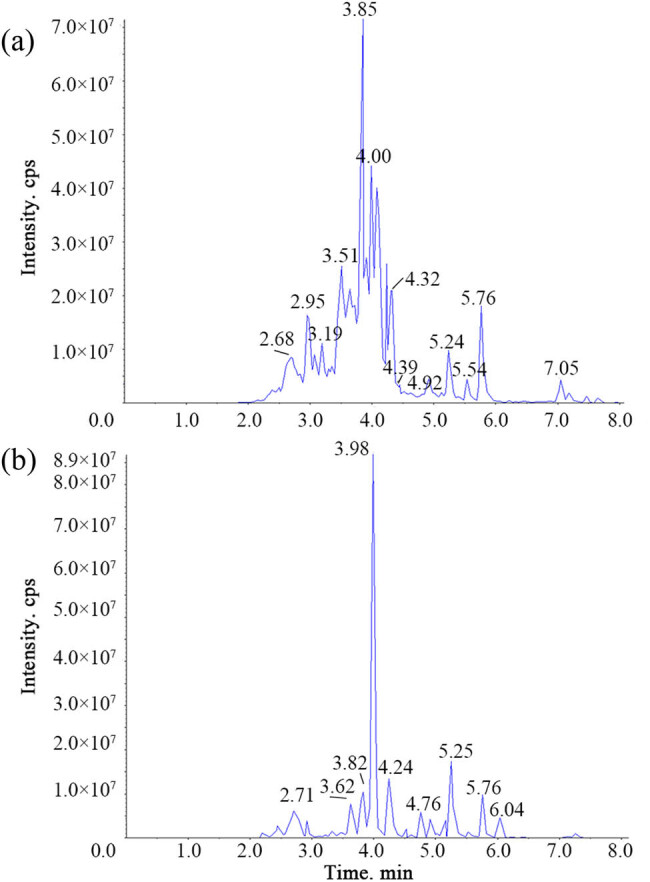
TIC diagrams of positive ion (a) and negative ion (b) in mass spectrometry of mixed samples.

**Figure 3:**
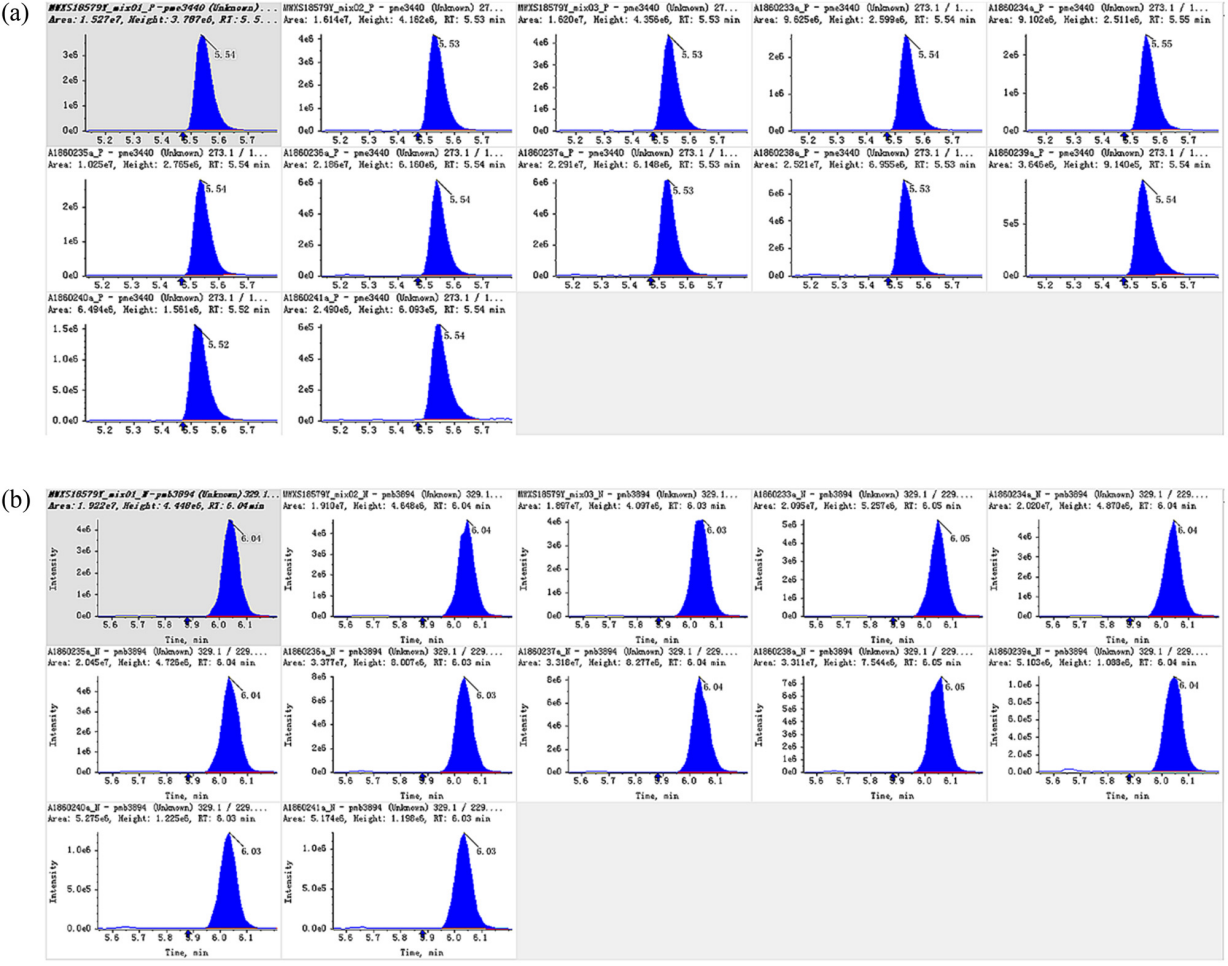
Integral correction diagram for quantitative analysis of positive ion (a) and negative ion (b) of randomly selected metabolites.

The mass spectrum data were processed by Software Analyst 1.6.3. Figure 2 shows the total ion flow diagram of the mixed sample. To compare the content difference of each metabolite in different samples, the mass spectrum analysis data of different samples were obtained by MRM, the peak area of all mass spectrum peaks was integrated, and the mass spectrum peaks of the same metabolite in different samples were integrated and corrected (Figure 3), to ensure the quality of the mixed sample. The abscissa is the retention time of metabolite detection, the ordinate is the ion current intensity of ion detection, and the peak area represents the relative content of the substance in the sample. A total of 130 flavonoid metabolites were identified and the data are shown in detail in Table S1.

### 3.3 Identification of metabolites

**Figure 4:**
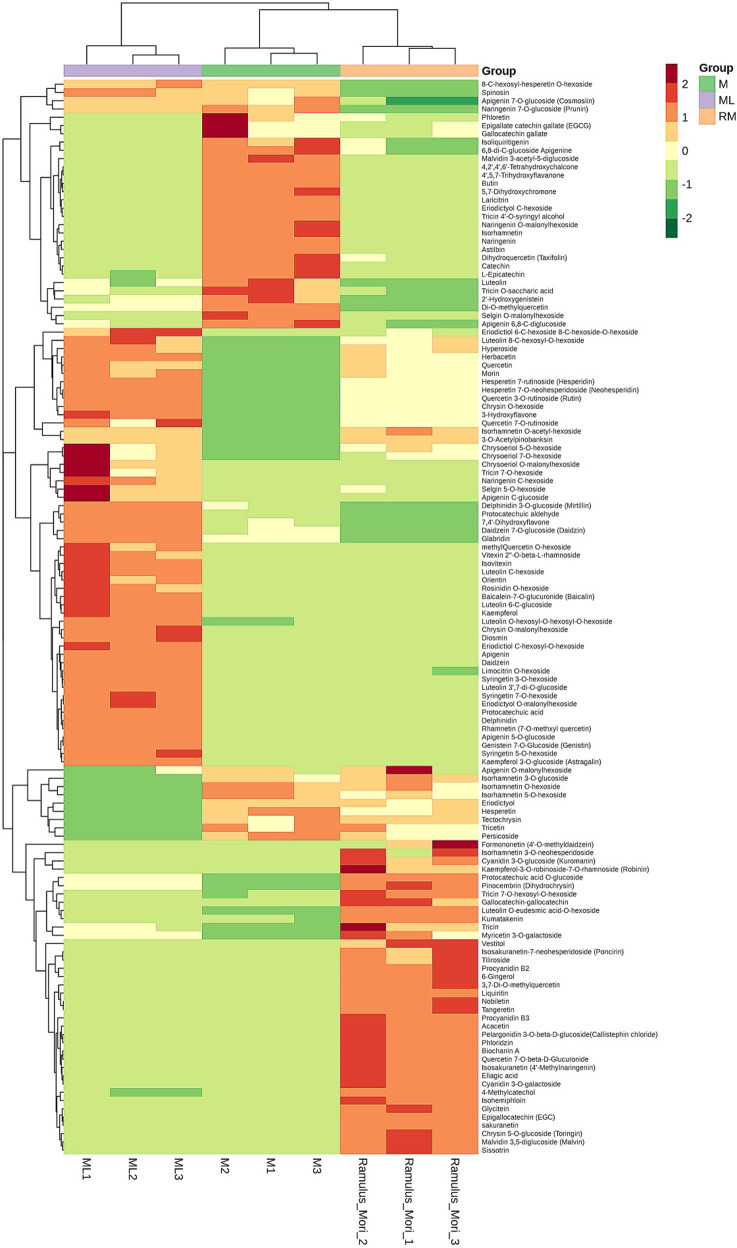
The overall cluster heat map of the sample. The metabolite content data were normalized by the extreme difference method. The transverse coordinates represented the sample name, the longitudinal coordinates represented the metabolite name, and the color represented the relative value (red for up-regulation, green for down-regulation).

The flavonoid metabolites of three parts of mulberry were analyzed by UPLC–MS/MS technology and database. A total of 130 metabolites were identified, including 47 flavone, 23 flavonols, 16 flavonoids, 8 anthocyanins, 8 isoflavone, 14 flavanone, 2 proanthocyanidins, and 12 polyphenols (Table S1). The range method was used to normalize the data of metabolite content, and the R software was used to cluster the accumulation patterns of metabolites in different samples. As shown in Figure 4, we can see that there are obvious differences in the content of flavonoid metabolites among ML, M, and RM. Compared with M, the contents of most flavonoid metabolites in ML and RM were up-regulated. Compared with ML, the contents of flavone and flavonol metabolites in RM and m were down-regulated, and each tissue had a uniquely high content of flavonoid metabolites.

### 3.4 Differential flavonoid metabolite analysis based on PCA

**Figure 5:**
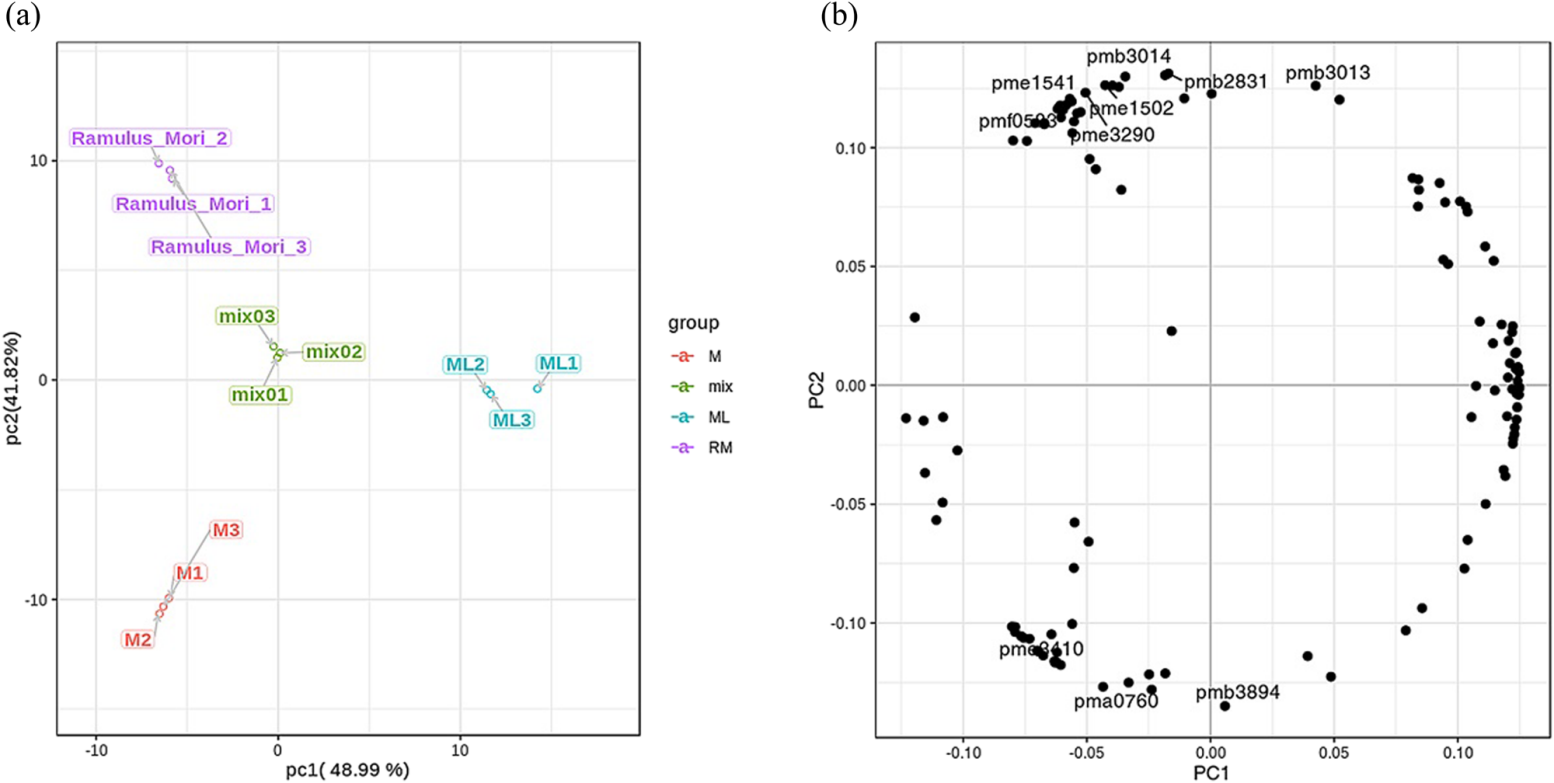
Differential flavonoid metabolite analysis on the basis of (a) principal component (PCA) and (b) PCA loadings.

PCA uses several main components to reveal the internal structure between multiple variables. In this study, two main components, PC1 and PC2, were extracted and were 48.99% and 41.82%, respectively, and the cumulative contribution rate was 90.81%. In the PCA score diagram (Figure 5), M, RM, and ML were separated clearly, and the repeated samples were allocated closely together, which indicated that the experiment was repeatable and reliable. The obvious separation of M, mix (mixed QC group), ML, and RM showed that there was a great difference among the four groups.

### 3.5 Differential flavonoid metabolite analysis based on OPLS-DA

**Figure 6:**
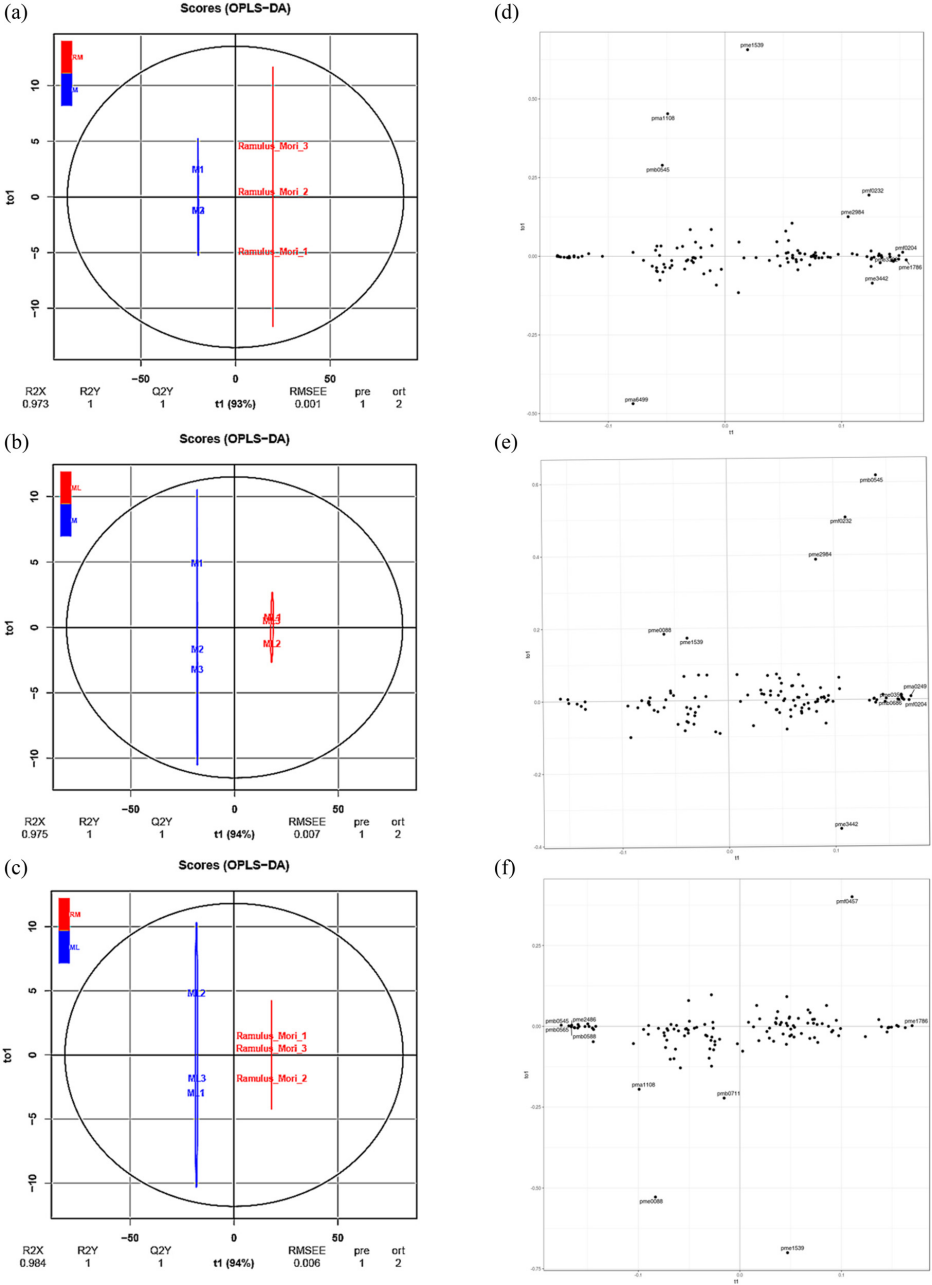
Identification analysis of flavonoid metabolites based on OPLS-DA. (a–c) OPLS-DA model plots for the comparison group M vs RM, ML vs M, and ML vs RM, respectively. (d–f) OPLS-DA loading plots for the comparison group M vs RM, ML vs M, and ML vs RM, respectively.

OPLS-DA, combined with orthogonal signal correction and PLS-DA, can decompose *X* matrix information into two kinds of information related to *Y* and unrelated. By removing unrelated differences, the different variables can be screened, which can maximize the difference between groups and is beneficial to the search for differential metabolites. The prediction parameters to evaluate the model were *R* ^2^ *X*, *R* ^2^ *Y*, and *Q* ^2^. Among them, *R* ^2^ *X* and *R* ^2^ *Y* represent the interpretation rate of the model to the *X* and *Y* matrices, respectively, and *Q* ^2^ represents the prediction ability of the model. The closer these three indexes are to 1, the more stable and reliable the model is. When *Q* ^2^ > 0.5, it can be considered an effective model, and *Q* ^2^ > 0.9 is an excellent model. In this study (Figure 6), the contents of flavonoid metabolites in the two groups were compared by the OPLS-DA model to evaluate M and RM (*R* ^2^ *X* = 0.973, *R* ^2^ *Y* = 1, *Q* ^2^ = 1), ML and M (*R* ^2^ *X* = 0.975, *R* ^2^ *Y* = 1, *Q* ^2^ = 1), and ML and RM (*R* ^2^ *X* = 0.984, *R* ^2^ *Y* = 1, *Q* ^2^ = 1). The *Q* ^2^ values of the three comparison groups were more than 0.9, which indicated that the model was stable and reliable and could be used to further screen different flavonoid metabolites.

### 3.6 Differential flavonoid metabolite screening, functional annotation, and enrichment analysis

Differential flavonoid metabolites were screened for each comparison group by combining the fold change and variable importance in project (VIP) values of the OPLS-DA model. The screening criteria were as follows: fold change ≥2 and fold change ≤0.5 and VIP ≥ 1. The screening results are shown in Table 3 and Table S2. From the screening results, we knew that there were 44 different metabolites of flavonoids between M and RM (29 up-regulated and 15 down-regulated). There were 38 between ML and M (9 up-regulated and 29 down-regulated) and 39 between ML and RM (18 up-regulated and 21 down-regulated). Most of the flavonoid metabolites were down-regulated in M and RM compared to ML, and most were also down-regulated in M compared to RM.

**Table 3:** The number of differential metabolites among groups

Group name	Total number of differences	Down-regulated	Up-regulated
M_vs_RM	44	15	29
ML_vs_M	38	29	9
ML_vs_RM	39	21	18

**Figure 7:**
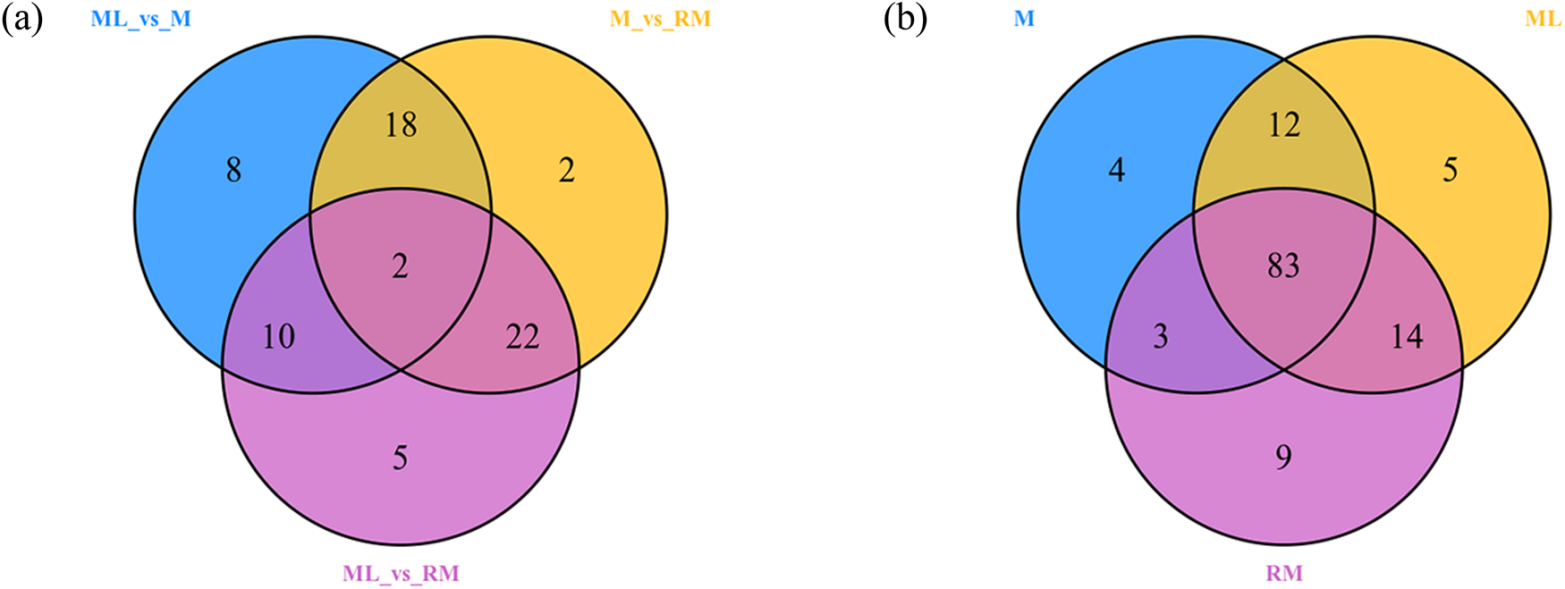
Venn diagram of differences in metabolites between groups (a) and among different medicinal parts (b).

After cross-comparison of differential metabolites in three comparison groups in the Venn diagram, two common differential metabolites, sissotrin and eriodictyol *O*-malonylhexoside, were observed (Figure 7a), and 18 common differential metabolites were observed among comparison groups M vs RM and ML vs M. Twenty-two common differential metabolites were observed among comparison groups M vs RM and ML vs RM, in the comparison group, ML vs M and ML vs RM had 10 common metabolites. The results showed that the flavonoid metabolites causing differences in M, RM, and ML were different. Through the comparative analysis of metabolites among the three tissue parts in the Venn diagram (Figure 7b), it was found that there were unique metabolites among each tissue site, which was consistent with the results of the previous clustering heat map.

**Figure 8:**
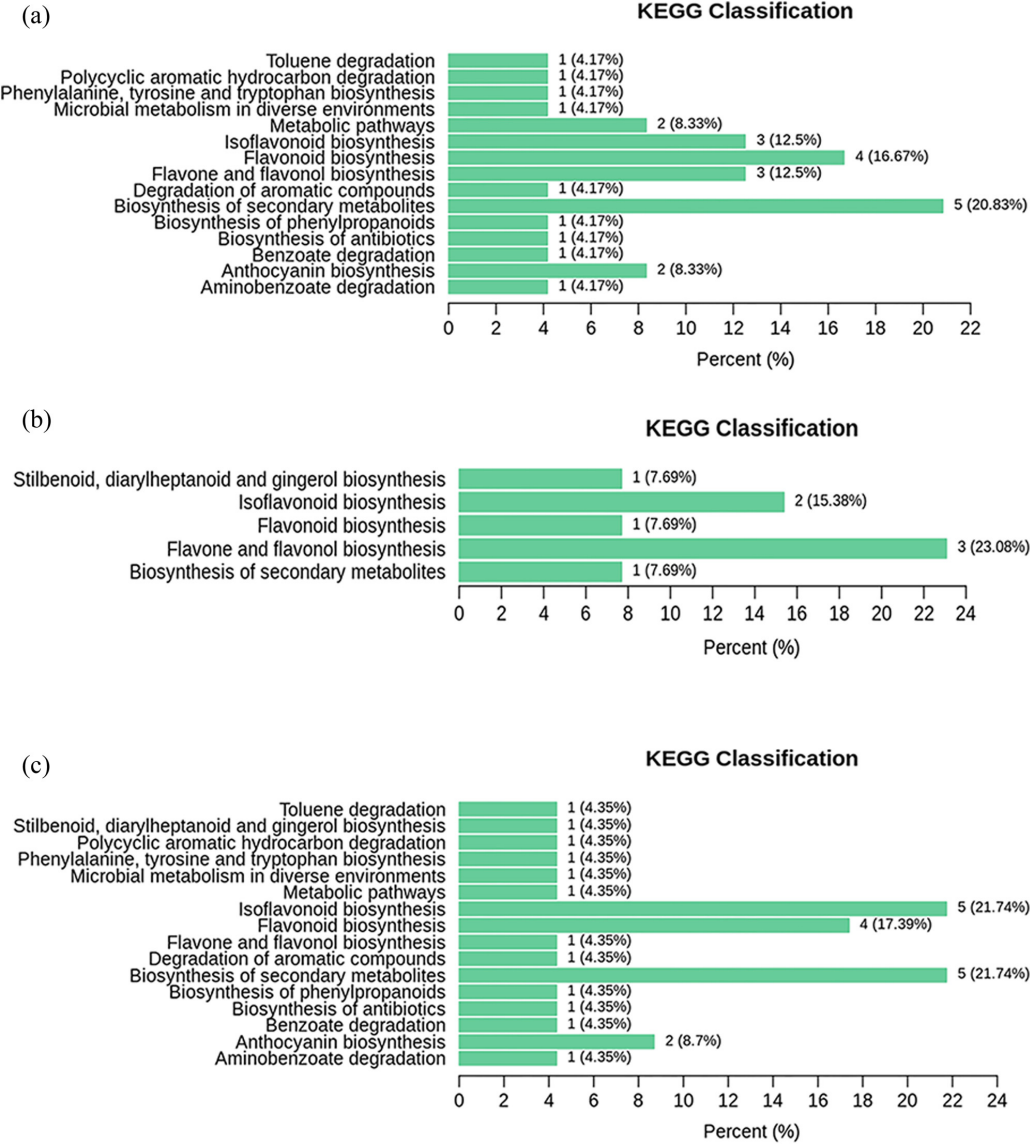
Comparison group M vs RM (a), ML vs M (b), and ML vs RM (c) differential metabolites KEGG classification.

Different metabolites interact with each other to form different pathways. The KEGG database was used to annotate and display the different metabolites, as shown in Figure 8. KEGG classification results showed that the different metabolites were mainly involved in the biosynthesis of flavonoids and flavonol, isoflavone biosynthesis, flavonoid biosynthesis, and so on. Differential metabolites of M vs RM are mainly annotated and enriched in the biosynthesis of secondary metabolites, flavonoid biosynthesis, isoflavonoid biosynthesis, and flavone and flavonol biosynthesis. Differential metabolites of ML vs M are mainly annotated and enriched in flavone and flavonol biosynthesis and isoflavonoid biosynthesis. Differential metabolites of ML vs RM are mainly annotated and enriched in the biosynthesis of secondary metabolites, isoflavonoid biosynthesis, and flavonoid biosynthesis.

## 4 Discussion

Mulberry is a kind of common plant with medicinal and food value. Its leaves, branches, and fruits all have a hypoglycemic effect, but its clinical application is different, which may be caused by the difference in the content of some metabolites. In this study, the contents of flavonoid metabolites in three parts of mulberry were analyzed and compared. The results showed that there were significant differences in the content of flavonoid metabolites in the three parts. Compared with ML, most of the flavonoid metabolites of M and RM were down-regulated, and compared with RM, most of the flavonoid metabolites of m were down-regulated. The content of flavone in M was similar to that of the flavonol. According to the content of flavonoid, the medicinal value of ML and RM may be better than that of M. According to the enrichment results of KEGG, it is speculated that the functional differences may be caused by the content differences of flavone, flavonol, anthocyanins, and isoflavones.

### 4.1 Differential metabolites associated with flavonoids and flavonols

Compared with ML, the contents of flavone and flavonol in M and RM were lower. Rutin, a flavone, was identified in all samples, and there was no significant difference in content. Rutin can reduce cholesterol levels and reduce insulin resistance in diabetic mice through antioxidant stress and inflammatory reaction [10]. Luteolin was only identified in M and ML, and the content of M was higher than that of ML, but the content of its glycoside derivatives was generally lower than that of ML. Luteolin has good anti-inflammatory, antioxidant, and antidiabetic properties [11]. We found that 3′-oxymethyl myricetin was only identified in M. Myricetin and its glycoside derivatives had antioxidant, anti-inflammatory, and anti-diabetes activities, and it showed stronger antioxidant and anti-diabetes activities than other flavonoids [12]. There was no significant difference between the three parts. Astragaloside can regulate blood glucose and improve the antioxidant capacity of the body [13,14]. Quercetin has no significant difference in all three tissue parts, but its glycoside derivatives have differences. Quercetin and its derivatives have a variety of biological activities such as anti-cancer, hypoglycemic, anti-inflammatory, antioxidant, and so on [15].

### 4.2 Differential metabolites associated with anthocyanins

Anthocyanin is a kind of plant pigment, with a strong antioxidant function, which can eliminate free radicals and improve glucose and lipid metabolism, anti-inflammatory, and other effects [16]. Hu et al. study found that anthocyanins can ameliorate oxidative stress in the body and increase testosterone levels by inhibiting the production of reactive oxygen species to mitigate potential damage to mitochondrial membranes [17]. In Chen et al. study, anthocyanins reduced H_2_O_2_-induced oxidative stress in the retinal pigment epithelium by decreasing the level of oxidative products and increasing antioxidant enzyme activities, which could have a beneficial effect on age-related macular degeneration [18]. Similarly, anthocyanins can potentially benefit their associated diseases such as diabetes, obesity, inflammation, and cancer by ameliorating oxidative stress [19]. In this study, only geranium-3-*O*-glucoside and malvin were identified in RM. The contents of cyanidin galactoside and cyanidin-3-*O*-glucoside in RM were higher.

### 4.3 Differential metabolites associated with isoflavones

Isoflavone is ubiquitous in soybean. Fourteen isoflavones were detected in this study. It was worth noting that sissotrin was the only identified differential isoflavone metabolite among the three comparison groups. Sissotrin is only detected in ML and RM, but not in M. The content of RM is higher than that of ML, which has an antioxidant effect, but it has a weakening effect on glucose tolerance [20,21]. In this study, genistein was only identified in ML, which can inhibit angiogenesis and antioxidation and improve palmitic acid-induced insulin resistance in HepG2 cells [22].

The most abundant in M were 8-*C*-hexosyl-hesperetin *O*-hexoside and naringenin; the most abundant in ML were 8-*C*-hexosyl-hesperetin *O*-hexoside and astragalin; and the most abundant in RM were cyanidin 3-*O*-galactoside and gallocatechin–gallocatechin. Among the different sites, 8-*C*-hexosyl-hesperetin *O*-hexoside was the highest in ML, the next highest in M, and the lowest in RM; naringenin was the highest in M, the next highest in RM, and the lowest in ML; astragalin was the highest in ML, second in RM, and the lowest in M; cyanidin 3-*O*-galactoside and gallocatechin–gallocatechin were the highest in RM, second in ML, and the lowest in M. Lee et al. investigated the leaf and fruit contents of Korean mulberry variety Baekokwang flavonoid content, and in agreement with this study, the content was higher in ML than in M [23]. The reason for the differences in flavonoid content in different parts of the plant may be the differences in the expression of flavonoid-related genes [24]. Xu et al. studied the flavonoid biosynthesis genes in mulberry leaves at different harvest times and found that low temperatures may lead to an increase in flavonoid content by increasing the expression of flavonoid biosynthesis-related genes [25]. Similarly, the flavonoid content varied even in the same parts of the same species of mulberry in some findings [26], which may also be due to the harvest time and environment of mulberry, among others, influencing the expression of flavonoid-related genes. Although these factors may affect the content of flavonoid components to a certain extent by influencing gene expression, different parts of mulberry do have different medicinal properties, and the present study to explore the differences in flavonoid components of different parts of mulberry is an important revelation for a deeper understanding and guidance of mulberry application.

Some studies have shown that diabetes is associated with antioxidant activity, inflammation, as well as glycolipid metabolism, and the content differences of flavonoid metabolites in each site mainly involve flavone, flavonol, anthocyanins, isoflavone metabolic pathways, and the metabolites with differences in each pathway also show different degrees of antidiabetic activity, and the content of flavonoid metabolites in each site ML is comparable to that in RM and generally higher than that in M. Therefore, it was inferred from this plant metabolome that mulberry leaves and mulberry branches may be superior to mulberry fruits in their antidiabetic activities, and the reason for the difference in activities may result from the difference in flavone, flavonol, anthocyanins, and isoflavone contents. Subsequent studies can extract the effective sites at each site separately for experimental comparison and further verify this inference.

## Supplementary Material

Supplementary Table
