# Supplementary material for: Differences in the flavonoid composition of the leaves, fruits, and branches of mulberry are distinguished based on a plant metabolomics approach
Source: Open Life Sci. 2024 Jun 27;19(1):20220886. doi: 10.1515/biol-2022-0886 (PMC11211874; doi:10.1515/biol-2022-0886)
Supplement: Supplementary Table [file biol-2022-0886-sm.pdf]

Supplementary material

Table S1: Total flavonoid metabolites identified in leaves, fruits and branches of mulberry

Index	Q1 (Da)	Q3 (Da)	Rt (min)	Molecular weight (Da)	Ionization model	KEGG ID	Compounds	Class
pma0214	479.00	317.00	3.91	478.00	[M+H]+	—	methylQuercetin *O*-hexoside	Flavonol
pma0249	479.00	317.00	3.52	478.00	[M+H]+	—	Selgin 5-*O*-hexoside	Flavone
pma0294	463.00	301.00	4.04	462.00	[M+H]+	—	Chrysoeriol 5-*O*-hexoside	Flavone
pma0724	435.00	285.00	3.79	434.00	[M+H]+	—	Naringenin *C*-hexoside	Flavone
pma0760	565.00	317.00	4.33	564.00	[M+H]+	—	Selgin *O*-malonylhexoside	Flavone
pma0791	521.00	273.00	4.50	520.00	[M+H]+	—	Naringenin *O*-malonylhexoside	Flavanone
pma0825	503.00	255.00	5.24	502.00	[M+H]+	—	Chrysin *O*-malonylhexoside	Flavone
pma1108	433.00	283.00	3.78	432.00	[M+H]+	—	Apigenin *C*-glucoside	Flavone
pma6199	417.00	255.00	4.99	416.00	[M+H]+	—	Chrysin *O*-hexoside	Flavone
pma6496	449.00	299.00	3.45	448.00	[M+H]+	—	Luteolin 6-*C*-glucoside	Flavone
pma6499	509.00	347.00	3.41	508.00	[M+H]+	—	Limocitrin *O*-hexoside	Flavone
pma6639	479.00	317.00	4.17	478.00	[M+H]+	—	Isorhamnetin *O*-hexoside	Flavonol
pmb0545	477.00	315.00	3.32	477.00	Protonated	—	Rosinidin *O*-hexoside	Anthocyanins
pmb0550	449.00	287.00	2.59	449.00	Protonated	C08604	Cyanidin 3-*O*-glucoside (Kuromanin)	Anthocyanins
pmb0565	509.00	347.00	3.41	508.00	[M+H]+	—	Syringetin 3-*O*-hexoside	Flavonol
pmb0569	509.00	347.00	4.17	508.00	[M+H]+	—	Syringetin 5-*O*-hexoside	Flavone
pmb0576	519.00	271.00	4.23	518.00	[M+H]+	—	Apigenin *O*-malonylhexoside	Flavone
pmb0580	417.00	255.00	5.00	416.00	[M+H]+	—	Chrysin 5-*O*-glucoside (Toringin)	Flavone
pmb0588	611.00	449.00	3.32	610.00	[M+H]+	—	"Luteolin 3′,7-di-*O*-glucoside"	Flavone
pmb0595	479.00	317.00	4.17	478.00	[M+H]+	—	Isorhamnetin 5-*O*-hexoside	Flavonol
pmb0602	509.00	347.00	4.17	508.00	[M+H]+	—	Syringetin 7-*O*-hexoside	Flavone
pmb0607	463.00	301.00	4.30	462.00	[M+H]+	—	Chrysoeriol 7-*O*-hexoside	Flavone
pmb0608	549.00	301.00	4.55	548.00	[M+H]+	—	Chrysoeriol *O*-malonylhexoside	Flavone
pmb0618	627.00	465.00	2.78	626.00	[M+H]+	—	8-*C*-hexosyl-hesperetin *O*-hexoside	Flavone
pmb0619	775.00	613.00	2.67	774.00	[M+H]+	—	Eriodictiol 6-*C*-hexoside 8-*C*-hexoside-*O*-hexoside	Flavone
pmb0628	613.00	451.00	3.09	612.00	[M+H]+	—	Eriodictiol *C*-hexosyl-*O*-hexoside	Flavone
pmb0644	449.00	299.00	3.45	448.00	[M+H]+	—	Luteolin *C*-hexoside	Flavone
pmb0665	611.00	465.00	3.78	610.00	[M+H]+	—	Luteolin 8-*C*-hexosyl-*O*-hexoside	Flavone
pmb0686	537.00	289.00	4.41	536.00	[M+H]+	—	Eriodictyol *O*-malonylhexoside	Flavanone
pmb0711	611.00	303.00	3.70	610.00	[M+H]+	—	Quercetin 7-*O*-rutinoside	Flavonol

(Continued)

Table S1: *Continued*

Index	Q1 (Da)	Q3 (Da)	Rt (min)	Molecular weight (Da)	Ionization model	KEGG ID	Compounds	Class
pmb0713	655.00	331.00	3.37	654.00	[M+H]+	—	Tricin 7-*O*-hexosyl-*O*-hexoside	Flavone
pmb0736	493.00	331.00	4.30	492.00	[M+H]+	—	Tricin 7-*O*-hexoside	Flavone
pmb0835	611.00	287.00	2.36	610.00	[M+H]+	—	Gallocatechin-gallocatechin	Polyphenol
pmb2831	315.00	153.00	2.44	316.00	[M−H]−	—	Protocatechuic acid *O*-glucoside	Polyphenol
pmb2850	329.00	314.00	5.73	330.00	[M−H]−	—	Tricin	Flavone
pmb2954	771.00	609.00	2.83	772.00	[M−H]−	—	Luteolin *O*-hexosyl-*O*-hexosyl-*O*-hexoside	Flavone
pmb3006	431.00	269.00	4.21	432.00	[M−H]−	C04608	Apigenin 7-*O*-glucoside (Cosmosiin)	Flavone
pmb3013	519.00	314.00	4.32	520.00	[M−H]−	—	Isorhamnetin *O*-acetyl-hexoside	Flavonol
pmb3014	641.00	285.00	4.67	642.00	[M−H]−	—	Luteolin *O*-eudesmic acid-*O*-hexoside	Flavone
pmb3023	449.00	287.00	3.37	450.00	[M−H]−	—	Eriodictyol *C*-hexoside	Flavone
pmb3041	521.00	329.00	3.81	522.00	[M−H]−	—	Tricin *O*-saccharic acid	Flavone
pmb3051	495.00	329.00	5.75	496.00	[M−H]−	—	Tricin 4′-*O*-syringyl alcohol	Flavone
pmb3894	329.00	229.00	5.91	330.00	[M−H]−	—	Di-*O*-methylquercetin	Flavonol
pme0001	609.00	301.00	4.07	610.00	[M−H]−	C09806	Hesperetin 7-*O*-neohesperidoside (Neohesperidin)	Flavanone
pme0088	285.00	151.00	5.00	286.00	[M−H]−	C01514	Luteolin	Flavone
pme0197	609.00	301.00	3.70	610.00	[M−H]−	C05625	Quercetin 3-*O*-rutinoside (Rutin)	Flavonol
pme0199	301.00	151.00	5.12	302.00	[M−H]−	C00389	Quercetin	Flavonol
pme0200	287.00	153.00	5.73	286.00	[M+H]+	C05903	Kaempferol	Flavonol
pme0201	291.00	139.00	2.99	290.00	[M+H]+	C06562	Catechin	Polyphenol
pme0355	255.00	137.00	4.97	254.00	[M+H]+	C10208	Daidzein	Isoflavone
pme0359	433.00	271.00	3.81	432.00	[M+H]+	—	Apigenin 5-*O*-glucoside	Flavone
pme0372	435.00	273.00	4.22	434.00	[M+H]+	C09099	Naringenin 7-*O*-glucoside (Prunin)	Flavanone
pme0377	273.00	153.00	5.59	272.00	[M+H]+	C00509	Naringenin	Flavanone
pme0379	271.00	153.00	5.63	270.00	[M+H]+	C01477	Apigenin	Flavone
pme0426	123.00	108.00	4.05	124.00	[M−H]−	C06730	4-Methylcatechol	Polyphenol
pme0434	577.00	407.00	3.03	578.00	[M−H]−	—	Procyanidin B2	Proanthocyanidins
pme0436	577.00	407.00	2.79	578.00	[M−H]−	—	Procyanidin B3	Proanthocyanidins
pme0442	303.00	229.00	2.98	303.00	Protonated	C05908	Delphinidin	Anthocyanins
pme0460	291.00	139.00	3.32	290.00	[M+H]+	C09727	L-Epicatechin	Polyphenol
pme1201	273.00	167.00	5.56	274.00	[M−H]−	C00774	Phloretin	Flavanone
pme1398	465.00	303.00	2.26	465.00	Protonated	C12138	Delphinidin 3-*O*-glucoside (Mirtillin)	Anthocyanins
pme1486	457.00	169.00	3.33	458.00	[M−H]−	C09731	Epigallate catechin gallate (EGCG)	Polyphenol
pme1498	269.00	237.00	6.33	268.00	[M+H]+	C00858	Formononetin (4′-*O*-methyldaidzein)	Isoflavone
pme1502	315.00	300.00	7.23	314.00	[M+H]+	—	Kumatakenin	Flavonol
pme1514	305.00	125.00	2.76	306.00	[M−H]−	C12136	Epigallocatechin (EGC)	Polyphenol

Table S1: *Continued*

Index	Q1 (Da)	Q3 (Da)	Rt (min)	Molecular weight (Da)	Ionization model	KEGG ID	Compounds	Class
pme1518	403.00	373.00	7.06	402.00	[M+H]+	C10112	Nobiletin	Flavone
pme1524	305.00	231.00	4.14	304.00	[M+H]+	C01617	Dihydroquercetin (Taxifolin)	Flavonol
pme1539	623.00	314.00	3.66	624.00	[M−H]−	—	Isorhamnetin 3-*O*-neohesperidoside	Flavonol
pme1541	283.00	268.00	7.06	284.00	[M−H]−	C01470	Acacetin	Flavone
pme1547	445.00	269.00	4.09	446.00	[M−H]−	C10025	Baicalein-7-*O*-glucuronide (Baicalin)	Flavone
pme1550	373.00	343.00	7.54	372.00	[M+H]+	C10190	Tangeretin	Flavone
pme1580	287.00	135.00	5.05	288.00	[M−H]−	C05631	Eriodictyol	Flavanone
pme1587	417.00	255.00	3.36	416.00	[M+H]+	C10216	Daidzein 7-*O*-glucoside (Daidzin)	Isoflavone
pme1590	317.00	153.00	5.87	316.00	[M+H]+	C10084	Isorhamnetin	Flavonol
pme1611	433.00	313.00	3.75	434.00	[M−H]−	—	Isohemiphloin	Flavone
pme1622	447.00	285.00	3.93	448.00	[M−H]−	C12249	Kaempferol 3-*O*-glucoside (Astragalin)	Flavonol
pme1624	433.00	283.00	3.78	432.00	[M+H]+	C01714	Isovitexin	Flavone
pme1662	287.00	167.00	6.96	286.00	[M+H]+	C09833	sakuranetin	Flavone
pme1786	655.00	331.00	2.61	655.00	Protonated	C08718	"Malvidin 3,5-diglucoside (Malvin)"	Anthocyanins
pme2247	303.00	285.00	6.57	302.00	[M+H]+	C10788	Ellagic acid	Polyphenol
pme2321	303.00	153.00	5.75	302.00	[M+H]+	C01709	Hesperetin	Flavanone
pme2478	137.00	108.00	3.06	138.00	[M−H]−	C16700	Protocatechuic aldehyde	Polyphenol
pme2486	155.00	109.00	2.31	154.00	[M+H]+	C00230	Protocatechuic acid	Polyphenol
pme2949	609.00	301.00	4.08	610.00	[M−H]−	C09755	Hesperetin 7-rutinoside (Hesperidin)	Flavanone
pme2982	257.00	153.00	7.05	256.00	[M+H]+	C09827	Pinocembrin (Dihydrochrysin)	Flavanone
pme2984	593.00	285.00	5.09	594.00	[M−H]−	C09830	Isosakuranetin-7-neohesperidoside (Poncirin)	Flavanone
pme3134	237.00	209.00	7.73	238.00	[M−H]−	C20871	3-Hydroxyflavone	Flavonol
pme3210	433.00	271.00	4.01	432.00	[M+H]+	C09126	Genistein 7-*O*-Glucoside (Genistin)	Isoflavone
pme3217	257.00	137.00	6.09	256.00	[M+H]+	C08650	Isoliquiritigenin	Flavanone
pme3227	579.00	433.00	3.68	578.00	[M+H]+	C12628	Vitexin 2″-*O*-beta-L-rhamnoside	Flavone
pme3250	285.00	270.00	7.00	284.00	[M+H]+	C00814	Biochanin A	Isoflavone
pme3251	283.00	268.00	5.13	284.00	[M−H]−	C14536	Glycitein	Isoflavone
pme3276	285.00	217.00	4.89	286.00	[M−H]−	C12134	2′-Hydroxygenistein	Isoflavone
pme3290	331.00	316.00	6.63	330.00	[M+H]+	C01265	"3,7-Di-*O*-methylquercetin"	Flavonol
pme3303	303.00	257.00	4.51	302.00	[M+H]+	C10192	Tricetin	Flavone
pme3369	315.00	165.00	6.43	316.00	[M−H]−	C10176	Rhamnetin (7-*O*-methxyl quercetin)	Flavonol
pme3392	433.00	271.00	2.83	433.00	Protonated	—	Pelargonidin 3-*O*-beta-D-glucoside （Callistephin chloride)	Anthocyanins
pme3400	447.00	285.00	5.16	446.00	[M+H]+	C05376	Sissotrin	Isoflavone

(Continued)

Table S1: *Continued*

Index	Q1 (Da)	Q3 (Da)	Rt (min)	Molecular weight (Da)	Ionization model	KEGG ID	Compounds	Class
pme3410	333.00	153.00	5.15	332.00	[M+H]+	C12633	Laricitrin	Flavonol
pme3442	477.00	301.00	3.76	478.00	[M−H]−	—	Quercetin 7-*O*-β-D-Glucuronide	Flavonol
pme3464	285.00	164.00	6.98	286.00	[M−H]−	C05334	Isosakuranetin (4′-Methylnaringenin)	Flavanone
pme3468	739.00	593.00	3.35	740.00	[M−H]−	C10178	Kaempferol-3-O-robinoside-7-*O*-rhamnoside (Robinin)	Flavonol
pme3473	271.00	151.00	5.59	272.00	[M−H]−	C09614	Butin	Flavone
pme3484	479.00	316.00	3.53	480.00	[M−H]−	—	Myricetin 3-*O*-galactoside	Flavonol
pme3507	253.00	117.00	4.75	254.00	[M−H]−	C12123	"7,4′-Dihydroxyflavone"	Flavone
pme3514	301.00	151.00	5.11	302.00	[M−H]−	C10105	Morin	Flavonol
pmf0011	593.00	473.00	3.19	594.00	[M−H]−	—	"Apigenin 6,8-*C*-diglucoside"	Flavone
pmf0012	595.00	457.00	3.18	594.00	[M+H]+	—	"6,8-di-*C*-glucoside Apigenine"	Flavone
pmf0027	449.00	287.00	2.68	448.00	[M]+	C08647	Cyanidin 3-*O*-galactoside	Anthocyanins
pmf0057	271.00	151.00	5.66	272.00	[M−H]−	C06561	"4,2′,4′,6′-Tetrahydroxychalcone "	Flavone
pmf0058	271.00	151.00	5.66	272.00	[M−H]−	C00509	"4′,5,7-Trihydroxyflavanone"	Flavanone
pmf0109	313.00	253.00	7.13	314.00	[M−H]−	—	3-*O*-Acetylpinobanksin	Flavonoid
pmf0113	271.00	135.00	6.27	272.00	[M−H]−	C16225	Vestitol	Flavonoid
pmf0204	465.00	303.00	3.73	464.00	[M+H]+	—	Hyperoside	Flavonoid
pmf0232	593.00	285.00	4.92	594.00	[M−H]−	C17140	Tiliroside	Flavonoid
pmf0247	449.00	299.00	3.46	448.00	[M+H]+	C10114	Orientin	Flavonoid
pmf0265	177.00	88.90	4.47	178.00	[M−H]−	C09001	"5,7-Dihydroxychromone"	Flavonoid
pmf0274	301.00	211.00	5.13	302.00	[M−H]−	—	Herbacetin	Flavonoid
pmf0330	607.00	445.00	3.67	608.00	[M−H]−	C17834	Spinosin	Flavonoid
pmf0360	449.00	151.00	3.97	450.00	[M−H]−	C17449	Astilbin	Flavonoid
pmf0369	477.00	315.00	4.54	478.00	[M−H]−	—	Persicoside	Flavonoid
pmf0375	479.00	317.00	4.18	478.00	[M+H]+	—	Isorhamnetin 3-*O*-glucoside	Flavonoid
pmf0379	457.00	169.00	3.44	458.00	[M−H]−	—	Gallocatechin gallate	Polyphenol
pmf0457	293.00	99.10	7.10	294.00	[M−H]−	C10462	6-Gingerol	Polyphenol
pmf0549	607.00	299.00	4.14	608.00	[M−H]−	C10039	Diosmin	Flavonoid
pmf0551	269.00	226.00	8.57	268.00	[M+H]+	C11621	Tectochrysin	Flavonoid
pmf0569	417.00	255.00	3.87	418.00	[M−H]−	C16978	Liquiritin	Flavonoid
pmf0582	325.00	123.00	7.88	324.00	[M+H]+	C10421	Glabridin	Flavonoid
pmf0583	435.00	167.00	4.36	436.00	[M−H]−	C01604	Phloridzin	Flavonoid
pmf0616	697.00	535.00	3.03	697.00	Protonated	—	Malvidin 3-acetyl-5-diglucoside	Anthocyanins

Table S2: Differential flavonoid metabolites among leaves, fruits and branches of mulberry

M_Vs_RM			
**Index**	**Compounds**	**Class**	**Type**
pma0249	Selgin 5-*O*-hexoside	Flavone	Up
pma0294	Chrysoeriol 5-*O*-hexoside	Flavone	Up
pma0724	Naringenin *C*-hexoside	Flavone	Up
pma0760	Selgin *O*-malonylhexoside	Flavone	Down
pmb0550	Cyanidin 3-*O*-glucoside (Kuromanin)	Anthocyanins	Up
pmb0565	Syringetin 3-*O*-hexoside	Flavonol	Down
pmb0580	Chrysin 5-*O*-glucoside (Toringin)	Flavone	Up
pmb0588	Luteolin 3′,7-di-*O*-glucoside	Flavone	Down
pmb0607	Chrysoeriol 7-*O*-hexoside	Flavone	Up
pmb0619	Eriodictiol 6-*C*-hexoside 8-*C*-hexoside-*O*-hexoside	Flavone	Up
pmb0628	Eriodictiol *C*-hexosyl-*O*-hexoside	Flavone	Down
pmb0644	Luteolin *C*-hexoside	Flavone	Up
pmb0686	Eriodictyol *O*-malonylhexoside	Flavanone	Up
pmb2850	Tricin	Flavone	Up
pmb3014	Luteolin *O*-eudesmic acid-*O*-hexoside	Flavone	Up
pmb3051	Tricin 4′-*O*-syringyl alcohol	Flavone	Down
pme0088	Luteolin	Flavone	Down
pme0372	Naringenin 7-*O*-glucoside (Prunin)	Flavanone	Down
pme1398	Delphinidin 3-*O*-glucoside (Mirtillin)	Anthocyanins	Down
pme1514	Epigallocatechin (EGC)	Polyphenol	Up
pme1590	Isorhamnetin	Flavonol	Down
pme1786	Malvidin 3,5-diglucoside (Malvin)	Anthocyanins	Up
pme2247	Ellagic acid	Polyphenol	Up
pme2486	Protocatechuic acid	Polyphenol	Down
pme2984	Isosakuranetin-7-neohesperidoside (Poncirin)	Flavanone	Up
pme3251	Glycitein	Isoflavone	Up
pme3290	3,7-Di-*O*-methylquercetin	Flavonol	Up
pme3369	Rhamnetin (7-*O*-methxyl quercetin)	Flavonol	Down
pme3392	Pelargonidin 3-*O*-beta-D-glucoside (Callistephin chloride)	Anthocyanins	Up
pme3400	Sissotrin	Isoflavone	Up

(Continued)

Table S2: *Continued*

M_Vs_RM			
**Index**	**Compounds**	**Class**	**Type**
pme3410	Laricitrin	Flavonol	Down
pme3442	Quercetin 7-*O*-β-D-Glucuronide	Flavonol	Up
pme3468	Kaempferol-3-*O*-robinoside-7-*O*-rhamnoside (Robinin)	Flavonol	Up
pmf0027	Cyanidin 3-*O*-galactoside	Anthocyanins	Up
pmf0113	Vestitol	Flavonoid	Up
pmf0204	Hyperoside	Flavonoid	Up
pmf0232	Tiliroside	Flavonoid	Up
pmf0247	Orientin	Flavonoid	Up
pmf0274	Herbacetin	Flavonoid	Up
pmf0330	Spinosin	Flavonoid	Down
pmf0569	Liquiritin	Flavonoid	Up
pmf0582	Glabridin	Flavonoid	Down
pmf0583	Phloridzin	Flavonoid	Up
pmf0616	Malvidin 3-acetyl-5-diglucoside	Anthocyanins	Down
pma0249	Selgin 5-*O*-hexoside	Flavone	Down
pma0294	Chrysoeriol 5-*O*-hexoside	Flavone	Down
pma0724	Naringenin *C*-hexoside	Flavone	Down
pma6199	Chrysin *O*-hexoside	Flavone	Down
pma6496	Luteolin 6-*C*-glucoside	Flavone	Down
pmb0545	Rosinidin *O*-hexoside	Anthocyanins	Down
pmb0569	Syringetin 5-*O*-hexoside	Flavone	Down
pmb0602	Syringetin 7-*O*-hexoside	Flavone	Down
pmb0607	Chrysoeriol 7-*O*-hexoside	Flavone	Down
pmb0608	Chrysoeriol *O*-malonylhexoside	Flavone	Down
pmb0619	Eriodictiol 6-*C*-hexoside 8-*C*-hexoside-*O*-hexoside	Flavone	Down
pmb0644	Luteolin *C*-hexoside	Flavone	Down
pmb0665	Luteolin 8-*C*-hexosyl-*O*-hexoside	Flavone	Down
pmb0686	Eriodictyol *O*-malonylhexoside	Flavanone	Down
pmb0711	Quercetin 7-*O*-rutinoside	Flavonol	Down
pmb2850	Tricin	Flavone	Down
pmb3014	Luteolin *O*-eudesmic acid-*O*-hexoside	Flavone	Down
pmb3051	Tricin 4′-*O*-syringyl alcohol	Flavone	Up
pme0359	Apigenin 5-*O*-glucoside	Flavone	Down
pme1547	Baicalein-7-*O*-glucuronide (Baicalin)	Flavone	Down
pme1590	Isorhamnetin	Flavonol	Up

(Continued)

Table S2: *Continued*

M_Vs_RM			
**Index**	**Compounds**	**Class**	**Type**
pme1611	Isohemiphloin	Flavone	Up
pme2321	Hesperetin	Flavanone	Up
pme2949	Hesperetin 7-rutinoside (Hesperidin)	Flavanone	Down
pme3210	Genistein 7-*O*-Glucoside (Genistin)	Isoflavone	Down
pme3227	Vitexin 2″-*O*-beta-L-rhamnoside	Flavone	Down
pme3290	3,7-Di-*O*-methylquercetin	Flavonol	Down
pme3400	Sissotrin	Isoflavone	Down
pme3410	Laricitrin	Flavonol	Up
pme3442	Quercetin 7-*O*-β-D-Glucuronide	Flavonol	Down
pmf0204	Hyperoside	Flavonoid	Down
pmf0232	Tiliroside	Flavonoid	Down
pmf0247	Orientin	Flavonoid	Down
pmf0274	Herbacetin	Flavonoid	Down
pmf0360	Astilbin	Flavonoid	Up
pmf0369	Persicoside	Flavonoid	Up
pmf0457	6-Gingerol	Polyphenol	Up
pmf0616	Malvidin 3-acetyl-5-diglucoside	Anthocyanins	Up
pma0760	Selgin *O*-malonylhexoside	Flavone	Down
pma1108	Apigenin *C*-glucoside	Flavone	Down
pma6499	Limocitrin *O*-hexoside	Flavone	Down
pmb0545	Rosinidin *O*-hexoside	Anthocyanins	Down
pmb0550	Cyanidin 3-*O*-glucoside (Kuromanin)	Anthocyanins	Up
pmb0565	Syringetin 3-*O*-hexoside	Flavonol	Down
pmb0569	Syringetin 5-*O*-hexoside	Flavone	Down
pmb0580	Chrysin 5-*O*-glucoside (Toringin)	Flavone	Up
pmb0588	Luteolin 3',7-di-*O*-glucoside	Flavone	Down
pmb0602	Syringetin 7-*O*-hexoside	Flavone	Down
pmb0608	Chrysoeriol *O*-malonylhexoside	Flavone	Down
pmb0628	Eriodictiol *C*-hexosyl-*O*-hexoside	Flavone	Down
pmb0686	Eriodictyol *O*-malonylhexoside	Flavanone	Down
pme0359	Apigenin 5-*O*-glucoside	Flavone	Down
pme0372	Naringenin 7-*O*-glucoside (Prunin)	Flavanone	Down
pme1398	Delphinidin 3-*O*-glucoside (Mirtillin)	Anthocyanins	Down
pme1514	Epigallocatechin (EGC)	Polyphenol	Up

(Continued)

Table S2: *Continued*

M_Vs_RM			
**Index**	**Compounds**	**Class**	**Type**
pme1518	Nobiletin	Flavone	Up
pme1587	Daidzein 7-*O*-glucoside (Daidzin)	Isoflavone	Down
pme1611	Isohemiphloin	Flavone	Up
pme1624	Isovitexin	Flavone	Down
pme1786	Malvidin 3,5-diglucoside (Malvin)	Anthocyanins	Up
pme2247	Ellagic acid	Polyphenol	Up
pme2321	Hesperetin	Flavanone	Up
pme2486	Protocatechuic acid	Polyphenol	Down
pme3210	Genistein 7-*O*-Glucoside (Genistin)	Isoflavone	Down
pme3251	Glycitein	Isoflavone	Up
pme3369	Rhamnetin (7-*O*-methxyl quercetin)	Flavonol	Down
pme3392	Pelargonidin 3-*O*-beta-D-glucoside (Callistephin chloride)	Anthocyanins	Up
pme3400	Sissotrin	Isoflavone	Up
pme3468	Kaempferol-3-*O*-robinoside-7-*O*-rhamnoside (Robinin)	Flavonol	Up
pmf0027	Cyanidin 3-*O*-galactoside	Anthocyanins	Up
pmf0113	Vestitol	Flavonoid	Up
pmf0330	Spinosin	Flavonoid	Down
pmf0369	Persicoside	Flavonoid	Up
pmf0457	6-Gingerol	Polyphenol	Up
pmf0569	Liquiritin	Flavonoid	Up
pmf0582	Glabridin	Flavonoid	Down
pmf0583	Phloridzin	Flavonoid	Up
